# Investigating the Added Value of Beck’s Depression Inventory in Atherosclerosis Prediction: Lessons from Paracelsus 10,000

**DOI:** 10.3390/jcm13154492

**Published:** 2024-07-31

**Authors:** Christiane Dienhart, Elmar Aigner, Bernhard Iglseder, Vanessa Frey, Isabella Gostner, Patrick Langthaler, Bernhard Paulweber, Eugen Trinka, Bernhard Wernly

**Affiliations:** 1 Department of Internal Medicine I, Paracelsus Medical University, 5020 Salzburg, Austria; e.aigner@salk.at (E.A.); b.paulweber@salk.at (B.P.); 2 Department of Geriatric Medicine, Christian Doppler University Hospital, Paracelsus Medical University, 5020 Salzburg, Austria; b.iglseder@salk.at; 3 Department of Neurology, Christian Doppler University Hospital, Paracelsus Medical University and Centre for Cognitive Neuroscience, Affiliated Member of the European Reference Network EpiCARE, 5020 Salzburg, Austria; v.freyy@salk.at (V.F.); i.gostner@salk.at (I.G.); p.langthaler@salk.at (P.L.); e.trinka@salk.at (E.T.); 4 Department of Artificial Intelligence and Human Interfaces, Paris Lodron University of Salzburg, 5020 Salzburg, Austria; 5 Team Biostatistics and Big Medical Data, IDA Lab Salzburg, Paracelsus Medical University Salzburg, 5020 Salzburg, Austria; 6 Obesity Research Unit, Paracelsus Medical University, 5020 Salzburg, Austria; 7 Neuroscience Institute, Christian Doppler University Hospital, Paracelsus Medical University and Centre for Cognitive Neuroscience, Affiliated Member of the European Reference Network EpiCARE, 5020 Salzburg, Austria; 8 Department of Public Health, Health Services Research and Health Technology Assessment, UMIT—University for Health Sciences, Medical Informatics and Technology, 6060 Hall in Tirol, Austria; 9 Department of Internal Medicine I, Oberndorf Hospital, 5110 Salzburg, Austria; b.wernly@salk.at; 10 Institute for General and Preventive Medicine, Paracelsus Medical University, 5020 Salzburg, Austria

**Keywords:** brain health, cardiovascular disease, carotid doppler duplex, cerebrovascular disease, depression, mental health, plaque, SCORE2, stroke

## Abstract

**Background:** Depression is the most common mental illness worldwide and generates an enormous health and economic burden. Furthermore, it is known to be associated with an elevated risk of arteriosclerotic cardiovascular diseases (ASCVD), particularly stroke. However, it is not a factor reflected in many ASCVD risk models, including SCORE2. Thus, we analysed the relationship between depression, ASCVD and SCORE2 in our cohort. **Methods:** We analysed 9350 subjects from the Paracelsus 10,000 cohort, who underwent both a carotid artery ultrasound and completed a Beck Depression Inventory (BDI) screening. Patients were categorised binomially based on the BDI score. Atherosclerotic carotid plaque or absence was dichotomised for logistic regression modelling. Odds ratios and adjusted relative risks were calculated using Stata. **Results:** Subjects with an elevated BDI (≥14) had higher odds for carotid plaques compared to subjects with normal BDI, especially after adjusting for classical risk factors included in SCORE2 (1.21; 95%CI 1.03–1.43, *p* = 0.023). The adjusted relative risk for plaques was also increased (1.09; 95%CI 1.01–1.18, *p* = 0.021). Subgroup analysis showed an increased odds of plaques with increases in depressive symptoms, particularly in women and patients ≤55 yrs. **Conclusions:** In our cohort, the BDI score is associated with subclinical atherosclerosis beyond classical risk factors. Thus, depression might be an independent risk factor which may improve risk stratification if considered in ASCVD risk prediction models, such as SCORE2. Furthermore, reminding clinicians to take mental health into consideration to identify individuals at increased atherosclerosis risk may provide added opportunities to address measures which can reduce the risk of ASCVD.

## 1. Introduction

Depression is the most common mental illness in Europe, affecting over 40 million people annually in Europe and nearly 300 million worldwide. According to WHO data, it is among the largest single causes of disability worldwide, especially among women [1]. The prevalence in Europe varies between 6 and 10% depending on the method of measurement [2]. The cost of depression in Europe is substantial, with total costs reaching well over EUR 118 billion annually, largely driven by productivity losses as well as direct costs [3]. The cumulative global impact of depressive disorders in terms of lost economic output is estimated at approximately USD 1.15 trillion [4].

Atherosclerosis is a disease of the arterial vascular bed that contributes to the development of both cerebrovascular and cardiovascular diseases, which, according to WHO data, together contribute to the largest disease burden and commonest causes of death worldwide. Atherosclerotic cardiovascular disease (ASCVD) is the leading cause of morbidity and mortality worldwide, causing over one-third of deaths in the EU and costing over EUR 200 billion per year [5]. Stroke is the largest cause of death and disability among neurological diseases both in the EU and worldwide [6]. The Stroke Alliance for Europe estimated the direct and indirect costs of stroke care in Europe (2015) to be EUR 45 billion annually [7]. Total costs of stroke globally, including lost disability-adjusted life years, was estimated at USD 781 billion and is expected to rise exponentially [8].

The relationship between depression and ASCVD has been extensively studied, and a robust body of evidence suggests a bidirectional connection between the two [9,10,11,12,13,14,15]. The American Heart Association has issued a statement that depression should be considered a risk factor, especially for patients having suffered an acute coronary syndrome [16]. The British risk calculator, QRISK3, already takes into account both socioeconomic factors and mental health [17]. Thus, we believe that it is imperative that clinicians take non-classical factors into account not only to improve risk prediction models but also to increase awareness of possible opportunities to address and thus reduce the risk of ASCVD.

Although plaques on carotid doppler duplex (CDD) are, in general, associated with an increased risk of stroke, with high-grade stenosis (≥50%) even described as a direct cause of stroke [18,19], numerous studies have also shown plaques to be associated with increased cardiovascular risk [20,21,22,23]. Furthermore, CDD plaques have been evaluated for their predictive value in addition to classical risk factors within various populations and compared to risk models, including the Framingham model and SCORE. Here, it has recently been shown that a carotid plaque score might even outperform SCORE2 in predicting ASCVD risk [24,25].

Stroke is also commonly included in the definition of Major Adverse Cardiac Events (MACE)as a primary endpoint for cardiovascular trials [26]. Given the fluid associations between cardiovascular disease, stroke and ASCVD, with stroke in many cases being included in the wider definitions of cardiovascular disease, and both stroke and plaques applied as risk factors in popular ASCVD risk calculators [27,28,29,30], we feel confident in using CDD plaques as a proxy for atherosclerotic disease, in general, as well as for ASCVD in particular.

The SCORE2 model, updated in 2021, is established as the European Society of Cardiology’s model for quantifying the risk of ASCVD [31]. Like many models, it has particular merit at a population level but could benefit from adjustment on an individual level. It does not currently contain a mental health risk component. Similarly, as discussed by Wong et al. [30], we believe that risk factors that may be beneficial to modulating the SCORE2 model for a more personalised approach need to be evaluated, particularly with respect to gender. Additionally, addressing mental health and, particularly, depression in the context of ASCVD provides an enormous opportunity to potentially reduce ASCVD risk not only in optimising risk scores but in raising awareness of risk outside the classical factors. Furthermore, the addition of a mental health measure might encourage physicians outside of the classical internal medical and general practice specialities to address ASCVD risk, which may improve uptake and adherence to therapeutic measures, particularly given the enhanced therapeutic relationship often described in mental health care.

While in the US, a diagnosis of depression is included in discharge letters and medical correspondence to improve continuity of care, in Austria, psychiatric diagnoses are considered sensitive healthcare information and frequently not included in medical correspondence unless this has been explicitly approved by the patient [32]. Therefore, these diagnoses are often not recorded. Thus, we have explored the value of a point of care depression symptom measure, in this case, BDI, for its association with cardiovascular risk factors and SCORE2 using plaques found on carotid doppler duplex as a proxy. Our analysis includes both males and females within the cohort and considered separately.

## 2. Subjects and Methods

### 2.1. Subjects

The Paracelsus 10,000 study [33] is a prospective local population-based study in which a cohort of approximately 10,000 individuals aged 40 to 77 years from the Salzburg region were recruited randomly from a local population registry. Over 56,000 individuals were invited by letter, of which approximately one out of five responded. The entire data set was collected between 2013 and 2020 and has been analysed retrospectively. Follow-up visits began in autumn 2020 and are scheduled to be finished in 2026; these data are not included in this analysis. An in-depth description of the Paracelsus 10,000 study design, as well as additional demographic data, are detailed in the paper by Frey et al. [33].

During initial visits, most participants underwent an ultrasound examination of both carotid arteries. Ultrasounds of both carotid arteries, including measures of total plaque area (including both hard and soft plaques), were performed in a supine position using the same Panasonic GM-72P00A machine (Panasonic Healthcare, Yokohama, Japan) for all examinations, with at least 95% of examinations performed by the same experienced clinician. Plaques were defined as deposits on the vessel wall with a diameter of >1.5 mm, as well as an area >2.9 mm^2^. To increase accuracy, multiple measurements of each plaque from various transducer positions were made and averaged. The Gray-Weale score (types 1–4) was used to describe plaque morphology [34]. Stenosis was graded according to ECST guidelines [35]. The total plaque area was calculated as the sum of all plaque surfaces of the common carotid artery, the internal carotid artery (bulb and proximal course), and the external carotid artery of the respective side. The ultrasound images were recorded on the hospital imaging system and can be retrieved for future reference. The results were also collated in the Paracelsus 10,000 data bank for further analysis.

At baseline, participants also usually completed the 1996 version of Beck’s Depression Inventory (BDI). The BDI is a widely utilised self-report questionnaire designed to evaluate an individual’s severity of depressive symptoms. This subjective measure quantifies cognitive, emotional, and physical manifestations associated with depression. Respondents answer a series of questions or statements reflecting their feelings over a specified period, and the cumulative score provides an indication of the degree of depressive symptomatology. Covering diverse facets of depression, the inventory encompasses mood, self-perception, guilt, suicidal ideation, social withdrawal, and various physiological and cognitive aspects [36]. Although not developed as a diagnostic instrument, the BDI serves as a screening tool and facilitates the monitoring of changes in depressive symptoms over time. It is generally applied to complement clinical assessments for the evaluation and management of depression. The BDI employs a scoring system that yields an aggregate score reflective of the severity of depressive symptoms. The interpretation of these scores is categorised as minimal depression (0–13 points), indicative of minimal or absent depressive symptoms; mild depression (14–19 points), identifying the presence of mild depressive symptoms; moderate depression (20–28 points), suggestive of moderate levels of depressive symptoms; and severe depression (29–63 points), corresponding to the presence of severe depressive symptoms [36,37]. Using the BDI questionnaire, we categorised patients binomially depending on a BDI Score of either greater than or equal to 14 (‘depression’) or less than or equal to 13 (‘no depression’) for use in logistic regression analysis. We also evaluated the per-point association between the BDI Score and CDD pathologies.

The participants also completed questionnaires at the initial visit to assess educational status and net monthly household income, among others. Participants provided an estimate of their approximate monthly net household income in EUR: (1) ≤1000, (2) 1001–2000, (3) 2001–3000, (4) 3001–4000, (5) 4001–5000, (6) >5000, and (7) did not disclose [33]. Educational status was evaluated using the GISCED, as described by Schneider et al. [38,39].

**Figure 1:**
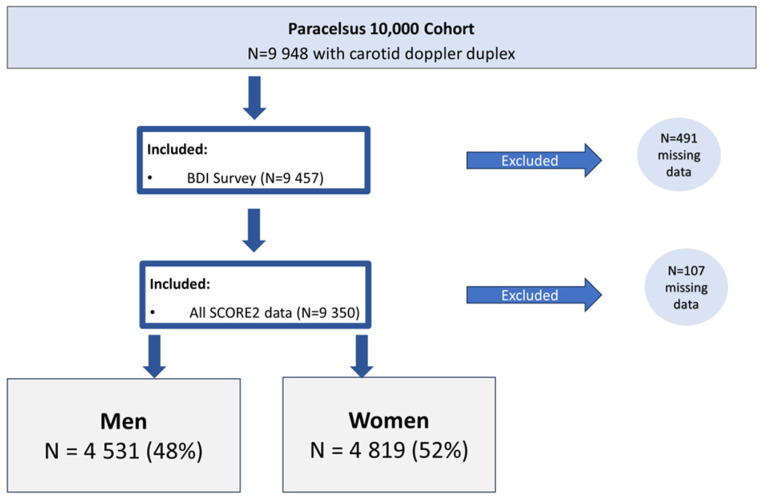
Inclusion and exclusion criteria (gender self-reported).

For our analysis, we included only subjects who had complete data available on Beck’s Depression Score, a full ultrasound analysis and all the data necessary to complete a SCORE2 calculation. See Figure 1 for details.

### 2.2. Statistical Analysis

Subjects were stratified by BDI score into those with minimal or no depression (BDI score ≤ 13) or at least a mild depression (BDI score ≥ 14) for further analysis. Numeric data were analysed and explained using median ± interquartile range (IQR) for continuous variables. The *p*-value was calculated using Student’s *t*-test. Categorical variables are expressed as number (N) and percentages (%) with chi-squared test with a significance level of *p* < 0.05 considered as significant.

To analyse the effects of the self-reported BDI score of depression symptoms on carotid pathology, we categorised plaques dichotomously as either present (1) or absent (0) as the dependent variable in the logistic regression models. Univariate and multivariable logistic regression were used to determine the relationship of carotid pathologies with the BDI score. We analysed several models using regression analysis: a baseline model examining the association between BDI and plaque formation (model 1), adjusted for age and sex (model 2), adjusted for age, sex, metabolic syndrome according to the IDF criteria, and educational status based on GISCED categories (model 3) and adjusted for SCORE2 components (model 4). Additionally, a final model (model 5) took into account components of model 3 as well as adjustments for the reported prescription of lipid-lowering medications. The choice of models reflects our clinical expertise, as well as our already published data [39] reflecting potential sources of endogeneity. Specifically, we examined whether any of our predictor variables could be influenced by the outcome variable or share common unobserved factors that might affect both the predictor and the outcome.

As the primary goal of our analysis was to illuminate the relationship between depressive symptoms and plaques in the Paracelsus 10,000 cohort, we performed a relative risk analysis on both unadjusted (crude) and adjusted models to take into account the added risk of depressive symptoms relative to classical risk factors as well as relative to SCORE2 risk calculator [40,41]. We used the script for SCORE2 for Stata provided by the authors of the SCORE2 working group, as described by Hagemann et al. [31].

Adjusted risk ratios (ARRs) provided a further tool to statistically analyse the association between the risk factor and a specific outcome (plaques) while simultaneously accounting for the influence of confounding variables. Beta coefficients were calculated using logistic regression. Adjusted risk ratios were calculated using the “adjrr” command in Stata, using these beta coefficients and ‘margins’ to produce estimates based on the beta coefficients [42]. We also calculated odds ratios (ORs) per point increase in BDI and the respective 95% confidence intervals (CIs). In addition, we performed a sensitivity analysis of the association for men versus women and for subjects >55 years or ≥55 years old.

All statistics were calculated using Stata Version 18 (StataCorp, College Station, TX, USA).

### 2.3. Ethics

The study was approved by the local ethics committee (Ethikkommission des Landes Salzburg, on 25 July 2012 (clinical trial number: 415-E/1521/6-2012). Informed consent documentation is available from all participants.

## 3. Results

Given the relapsing–remitting nature of depression and the tendency to avoid recording psychiatric diagnoses in medical correspondence in Austria, we have analysed the ability of a single-point application of a screening tool (BDI) to indicate an increased risk of plaque formation in our study population. We have also tried to elucidate associations among carotid plaques, BDI, and traditional risk factors. In addition, we analysed whether the addition of a ‘depression score’ using BDI could be additive to predictions based on the SCORE2 model. The analysis was performed for both men and women as well as for the group as a whole.

Overall, plaque levels in our population were recorded for men (47%) and women (30%) in the upper ranges compared to those shown in a previous meta-analysis by Song et al. [43]. However, levels did not reach the nearly 90% range described in a recent Norwegian study with a slightly older population (mean age 63.9 years) [24].

**Figure 2:**
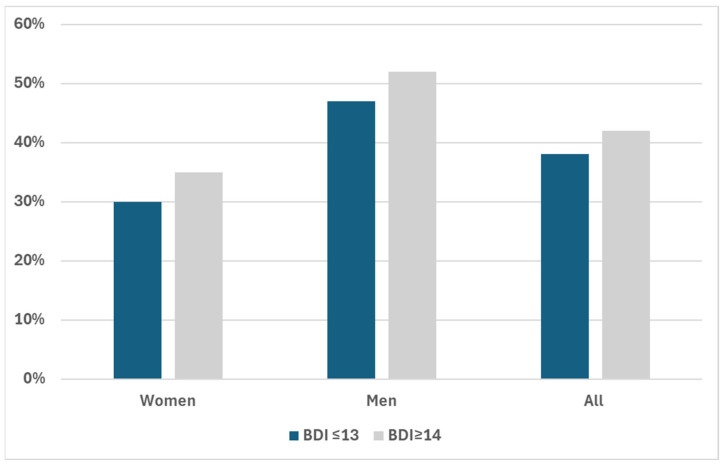
Proportion of patients with plaque based on BDI category.

Participants with a BDI score of 14 or greater (at least mild depressive symptoms) were more likely to have plaques on CDD examination than participants with a BDI score of 13 or less (Figure 2).

According to self-reported gender, men (48%) and women (52%) were nearly equally represented in our study population (N = 9350). However, women were over-represented (62%) in the BDI ≥ 14 group, in line with the understanding that female gender is associated with a greater risk of depression [44,45,46]. Furthermore, women with depressive symptoms were more likely to have plaques. Demographic details by self-reported gender are provided in Table 1 and Table 2 below.

In our study population, a higher BDI score is associated with components of metabolic syndrome, including higher triglycerides, abdominal circumference, and lower HDL. In addition, there was a higher incidence of pathological glucose tolerance with both a greater number of fasting glucose levels above 126 mg/dL (7.0 mmol/L) as well as a larger percentage of HbA1c above 6.5% (48 mmol/mol) in participants with a BDI ≥ 14. Average abdominal circumference also significantly increased with a higher BDI. Interestingly, there was no difference in LDL-cholesterol levels between the groups, although LDL-cholesterol levels were elevated well above recommended levels in our cohort in general. However, a chi-squared analysis of intake of lipid-lowering medication showed a statistically significant greater reported prescription of additional lipid-lowering medication; X2 (2, N = 9350) = 41.5087, *p* < 0.001. Elevated BDI was also associated with greater self-reported hypertension, dyslipidaemia, and type 2 diabetes mellitus (*p* < 0.001). There was, however, no difference in self-reported coronary artery disease nor congestive heart failure, perhaps either because participants were unaware of their coronary artery disease status and/or were chosen randomly from a ‘healthy’ collective, with an average age of 55 years, which is considered early for clinical manifestations of coronary artery disease but particularly, congestive heart failure [47,48,49]. Furthermore, there may be a certain level of unreported disease, particularly coronary artery disease [50].

Our data support previous reports of a high incidence of unhealthy behaviour, such as smoking, in patients with depressive symptoms [14,15,45]. Participants in the Paracelsus 10,000 study who recorded a higher BDI score were less likely to have never smoked. Additionally, subjects with BDI score ≥ 14 were more likely to be current smokers (27% of women and 26% of men reported smoking versus 17% of men and 18% of women with BDI ≤ 13; *p* < 0.001). Participants reporting greater depressive symptoms also self-reported a greater percentage of COPD in their medical history (3%; *p* < 0.001). Details are given in Table 1 and Table 2. According to our data, men reported a significantly higher percentage of alcohol abuse along with an elevated BDI, while in women, this relationship was not significant.

Participants in our cohort with higher BDI scores were also more likely to report lower income scores and lower levels of education. Studies suggest that both job satisfaction and job security are inversely related to depression, with unemployment, job stress, and inadequate employment increasing the risk for depression, especially in women [51,52].

Our logistic regression analysis shows a positive relationship between elevated depression symptoms and an increased risk of carotid plaques. After adjusting for age and sex, a BDI ≥ 14 was associated with a statistically significant overall higher likelihood of plaques in our population (OR 1.43; 95%CI 1.22–1.69, *p* < 0.001; ARR 1.18; 1.10–1.26, *p* < 0.001). Adjusting for age, sex, metabolic syndrome, and educational status (model 3), a BDI ≥ 14 increased the odds of plaque development (OR 1.32; 95%CI 1.11–1.56, *p* < 0.001; ARR 1.13; 1.05–1.21, *p* = 0.001. Adjusting model 3 for intake of lipid-lowering medication (referred to as model 5 in Table 3) still resulted in increased odds of plaque development with a BDI ≥ 14 (OR 1.25; 95%CI 1.06–1.49, *p* = 0.009; ARR 1.11; 1.03–1.19, *p* = 0.009). Furthermore, even after adjusting for SCORE2, an elevated BDI still showed higher odds of plaque presence (OR 1.21; 95%CI 1.03–1.42, *p* = 0.023; ARR 1.09; 1.01–1.18, *p* = 0.02). The full results of our logistic regression analysis can be seen in Table 3.

**Figure 3:**
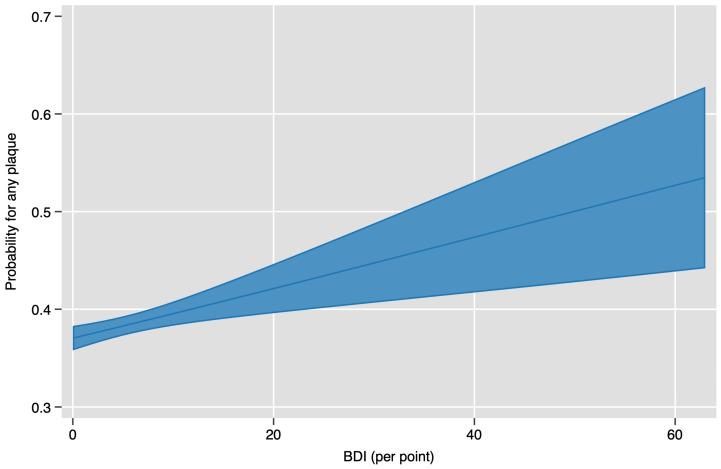
Plaque likelihood per point change in BDI.

Our data also indicate a potential per BDI point association between the risk of plaques and rising BDI score, as is seen in Figure 3.

Based on odds ratios, our data also signalled a slightly positive association per BDI point with the risk of plaque after adjusting for age and sex (model 2). However, the baseline association, as well as the overall association per point, after adjusting for SCORE2, were weak; see Table 4 below.

We also performed a sensitivity analysis, shown in Table 3, for participants based on self-declared sex. Here, our analysis tended to a higher likelihood of plaques in women who declared depressive symptoms when applying our baseline model (OR 1.28; 95%CI 1.06–1.54, *p* = 0.012). Furthermore, upon examining the baseline model by age group (participants over 55 years old versus participants up to and including 55 years of age), participants who were in the younger group showed a statistically significant increased risk for plaques (OR 1.46; 95%CI 1.17–1.81, *p* = 0.001) with a BDI ≥ 14.

Table 5 shows a summary of carotid pathologies found in our subjects. In women, there was a significant association between the presence of plaques and plaque area with BDI ≥ 14 (p0.012 and 0.023, respectively). In men, however, the plaque area was not significantly different between groups, although plaque presence was significant (*p* = 0.05).

## 4. Discussion

Addressing depression and ASCVD can help meet the WHO’s goals of ‘optimising brain health’, which ‘improves mental and physical health and also creates positive social and economic impacts, all of which contribute to greater well-being and help advance society’ [53]. Adding a depression risk measure to ASCVD risk assessment could further this goal.

The bi-directional association between depression and ASCVD has been consistently demonstrated, and thus, an association between plaques and BDI scores in our cohort is not altogether surprising. Numerous studies have linked depression as an independent risk factor for the development and progression of ASCVD, including coronary artery disease and stroke [9,13,14,15,54,55,56,57]. The reasons behind this association are various. The large increase in depression after major adverse cardiac events both underlines and complicates the analysis of this relationship.

Patients with depression and cardiovascular disease share common risky behaviours, such as smoking, sedentary lifestyle, poor diet, and obesity. In particular, it is estimated that nearly half of stroke risk could be attributed to these behavioural risk factors, while approximately 90% could be attributed to modifiable risk factors, including hypertension, obesity, diabetes, elevated lipids, and renal dysfunction [7,8]. Lifestyle factors affect modifiable risk factors for these conditions, particularly via the diabetes pathway, highlighting the complex interplay between mental health and ASCVD.

In our cohort, the association between smoking, obesity, and diabetic risk factors is shown, particularly for women. Furthermore, our subgroup analysis showed that depressive symptoms increased the likelihood of plaque formation, particularly in women.

Depression is also associated with alterations in hormonal regulation, particularly stimulation of the hypothalamic–pituitary–adrenal axis, which leads to increased levels of cortisol. Chronic exposure to elevated stress hormones can impact the cardiovascular system, contributing to hypertension, which is also a key risk factor for stroke [8,58,59]. Subjects within our cohort with an elevated BDI2 showed a statistically significant increase in self-reported hypertension.

Furthermore, it is thought that elevated levels of inflammatory markers observed in individuals with depression may also contribute to atherosclerosis [14,60]. In our population, we also found a statistically significant increase in hsCRP and leukocytes, which have been linked to inflammation in both depression and ASCVD [12,58,59].

Additionally, poor treatment adherence to blood pressure and lipid-lowering medications and suggested lifestyle interventions may lead to suboptimal management of ASCVD risk factors. In our cohort, there was no significant difference in LDL levels between groups of either reported gender, with BDI ≤ 13 and ≥14. However, a chi-squared analysis did show a statistically significant difference in reported lipid-lowering medication between the two groups, with subjects with a higher BDI score (≥14) reporting taking more lipid-lowering medication. Whether compliance is an issue or whether there are other factors playing a role is beyond the scope of this study. However, our research indicates that a quick, easy, and cheap single-point screening inventory, such as BDI, could add value for ASCVD risk prediction and should thus be considered for use in speciality clinics. Our data highlight an association between depression and carotid plaques in our middle-aged European cohort. Furthermore, our data analysis indicates a greater risk of plaques in women who declare depressive symptoms. As cardiovascular disease is still the leading cause of death (47% of all deaths) for women [60], the BDI might improve ASCVD risk assessment, particularly for women. Furthermore, our results suggest that a screening tool such as the BDI might be used to flag elevated risk in participants ≤55 years of age, who, along with women, are groups who tend to be overlooked as classical high-risk patients.

Furthermore, a depression score such as BDI may improve the SCORE2 risk model, particularly for females and patients under 55 years of age, who may not fall into the typical cardiovascular disease stereotype. However, additional research is needed in this area.

### Limitations

Although the association between an elevated BDI and plaques was highly significant, the absolute difference was only moderate. We expect that as both conditions are highly prevalent, our association is still clinically significant.

We have used a single-point evaluation of self-reported depressive symptoms from a screening tool, which is not intended to be diagnostic, as our proxy for depression. Screening tools, such as the BDI, tend to overestimate the presence of mental illness [61]. Furthermore, categorising participants binomially depending on having a BDI Score of either ≤13 (‘no depression’) or ≥14 (‘depression’) may also be criticised. However, the rate of depression (9.2%) measured by our method is not outside the ranges of depression reported in other Austrian and European studies during the data collection period [2,44,46,62,63]. Nevertheless, there is a large variation in results depending on the measurement tool, as well as differences in depression severity, persistence, and remissions. Given that depression has a variable persistence of around 18%, as well as remission rates varying between 12–60% [64], using actual DSM criteria (which we do not have available) might have substantially altered our results. In addition, depression rates may vary over time due to external pressures, and we are unable to judge how these may affect ASCVD rates. For example, depression rates in Austria varied dramatically during the COVID-19 lockdown from 4% prior to COVID-19 (2014) to 20% during the COVID-19 lockdown [63]. Clearly, additional research is needed to determine if a single-point depression screening tool such as BDI is truly the most effective measure of depression for its association with cardiovascular disease.

Furthermore, although the association between ASCVD and carotid plaques is reasonably robust and well documented, not all subjects who have pathology on carotid Doppler duplex will develop a MACE within the next ten years. However, we believe that our analysis is particularly interesting as it shows the possible value of a single-point analysis of depressive symptoms to measure the propensity for increased plaque development.

In addition, slightly less than half of the men in our cohort (47%) were found to have plaques, while among women, plaques were found in only 30%. Given that the average age of women in our population was near perimenopausal, this may affect the level of plaque development and also the level of reported depressive symptoms. It has been reported that women around menopausal age may experience increased depressive symptoms [65,66]. Thus, the effects of age and sex on the usefulness of a tool such as BDI need to be explored further.

Quantifying CDD plaques might also be an issue. However, we believe that our use of a binomial analysis, as well as the fact that most (>95%) of our CDD were performed by a single, experienced operator and using the same equipment, make our data more robust.

Finally, observational studies such as ours, by their nature, can indicate an association but not necessarily a cause.

Clearly, more research is necessary on the possible integration of a mental health/depression risk factor into the SCORE2 risk model and the value of the addition of single-point depressive symptom assessment, such as BDI, in speciality clinics.

## 5. Conclusions

Not only does our data support the association between depression and ASCVD risk, but it also indicates that adding a single-point depression symptom measure, such as BDI, may be satisfactory to evaluate this risk factor, at least in countries such as Austria, where depression may be underreported in medical documentation outside of the safe psychiatric space. Adding a depression measure to SCORE2 may also enhance the prediction of ASCVD risk, at least in some subgroups. A depression screen may be important, particularly for ASCVD risk assessment in women, who are also more likely to suffer from depression as a group, as well as die from cardiovascular disease. In addition, a depression indicator may also flag increased risk in younger individuals under 55 years of age, who might not fit the traditional ASCVD risk stereotype.

We believe that it is important to consider factors outside of the classical risk factors as these may improve ASCVD risk prediction for individuals. Additionally, by adding a mental health factor to the SCORE2 model, the interplay between two very important classes of disease can be better highlighted. Beyond this, in today’s era of increasing medical specialisation, underscoring the importance of mental health to an ASCVD risk model could encourage mental health teams to take advantage of treatment opportunities and support traditional prescribers in assuring treatment adherence and optimisation of a healthier lifestyle for patients at risk of ASCVD. Furthermore, applying a point of care depression score, such as BDI, in speciality clinics could encourage non-mental health specialists to address this issue with patients, improve continuity of care and reduce risk in patients who might otherwise struggle alone with their mental health issues.

## Figures and Tables

**Table 1:** Descriptive overview by self-reported gender (Men).

Men (N = 4531)	Total	BDI ≤ 13	BDI ≥ 14	*p*-Value
	N = 4531	N = 4200 (93%)	N = 331 (7%)	
Age (Median)	55 (50–62)	55 (50–62)	55 (50–61)	0.90
Age by decade				0.40
Age 40–49 yrs	24% (1073)	24% (1000)	22% (73)	
Age 50–59 yrs	44% (1974)	43% (1815)	48% (159)	
Age 60–69 yrs	28% (1281)	28% (1195)	26% (86)	
Age ≥ 70 yrs	4% (203)	5% (190)	4% (13)	
Total cholesterol mg/dL	206 (181–231)	206 (181–231)	206 (177–237)	0.93
Triglycerides mg/dL	111 (80–157)	110 (79–155)	131 (88–193)	<0.001
HDL cholesterol mg/dL	54 (45–64)	54 (46–64)	50 (42–61)	<0.001
LDL cholesterol mg/dL	142 (118–166)	142 (118–166)	138 (117–167)	0.51
Leucocytes	5.8 (4.9–6.9)	5.8 (4.9–6.9)	6.1 (5.0–7.7)	<0.001
hsCRP mg/dL	0.12 (0.07–0.23)	0.12 (0.06–0.22)	0.14 (0.07–0.28)	<0.001
Height cm	177 (173–182)	177 (173–182)	177 (172–181)	0.051
Weight kg	84 (77–94)	84 (76–93)	86 (78–97)	0.002
BMI kg/m^2^	27 (24–29)	27 (24–29)	28 (25–30)	<0.001
Obesity vs. Non-obese				<0.001
BMI < 30	79% (3573)	80% (3338)	71% (235)	
BMI ≥ 30	21% (951)	20% (856)	29% (95)	
Abdom. circumference cm	98 (91–105)	97 (91–105)	100 (93–110)	<0.001
Self-reported				
Dyslipidemia	14% (637)	14% (569)	21% (68)	<0.001
Diabetes Mellitus type 2	5% (219)	5% (189)	9% (30)	<0.001
Hypertension	27% (1193)	25% (1064)	40% (129)	<0.001
Coronary artery disease	3% (144)	3% (130)	4% (14)	0.24
Chronic heart failure	1% (34)	1% (29)	2% (5)	0.092
Peripheral vascular disease	1% (24)	0% (20)	1% (4)	0.074
COPD	2% (102)	2% (87)	5% (15)	0.003
Chronic kidney disease	1% (24)	0% (20)	1% (4)	0.075
Metabolic syndrome ^1^	21% (929)	20% (832)	30% (97)	<0.001
Diabetes Mellitus type 2	8% (357)	8% (316)	12% (41)	0.002
SCORE2 10-yr CVD risk (%)	6 (4–9)	6 (4–9)	6 (4–9)	0.008
HbA1c level (%)				0.003
HbA1c < 6.5%	96% (4216)	97% (3920)	93% (296)	
HbA1c ≥ 6.5%	4% (158)	3% (137)	7% (21)	
Glucose levels				0.014
Glucose < 126 mg/dL	94% (4250)	95% (3951)	91% (299)	
Glucose ≥ 126 mg/dL	6% (250)	5% (222)	9% (28)	
Alcohol g/week	63 (53–74)	63 (53–74)	65 (55–77)	0.022
Excessive alcohol intake ^2^	7% (255)	6% (225)	11% (30)	0.002
Smoking history				<0.001
Never smoker	42% (1905)	42% (1785)	36% (120)	
Previous smoking	40% (1825)	40% (1701)	37% (124)	
Current smoker	18% (801)	17% (714)	26% (87)	
Monthly household income				<0.001
≤EUR 1000	4% (161)	3% (126)	11% (35)	
EUR 1001–2000	22% (1001)	21% (896)	32% (105)	
EUR 2001–3000	30% (1346)	30% (1250)	29% (96)	
EUR 3001–4000	18% (824)	19% (785)	12% (39)	
EUR 4001–5000	11% (477)	11% (459)	5% (18)	
>EUR 5000	8% (370)	8% (353)	5% (17)	
Did not disclose	8% (352)	8% (331)	6% (21)	
GISCED educational status				<0.001
Low	6% (277)	6% (241)	11% (36)	
Medium	70% (3127)	70% (2901)	69% (226)	
High	24% (1079)	24% (1015)	20% (64)	

^1^ Metabolic syndrome according to International Diabetes Federation Criteria; ^2^ WHO Criteria.

**Table 2:** Descriptive overview by self-reported gender (Women).

Women (N = 4819)	Total	BDI ≤ 13	BDI ≥ 14	*p*-Value
	N = 4819	N = 4288 (89%)	N = 531 (11%)	
Age (Median)	54 (49–61)	55 (49–61)	54 (50–60)	0.18
Age by decade				0.005
Age 40–49	25% (1224)	26% (1095)	24% (129)	
Age 50–59	43% (2085)	42% (1822)	50% (263)	
Age 60–69	28% (1340)	28% (1222)	22% (118)	
Age ≥ 70	4% (170)	3% (149)	4% (21)	
Total cholesterol mg/dL	212 (188–238)	212 (188–238)	213 (188–238)	0.54
Triglycerides mg/dL	87 (65–120)	86 (65–118)	99 (75–138)	<0.001
HDL cholesterol mg/dL	70 (59–82)	70 (59–82)	65 (55–78)	<0.001
LDL cholesterol mg/dL	138 (114–163)	138 (114–163)	140 (117–166)	0.17
Leucocytes	5.7 (4.8–6.8)	5.7 (4.8–6.8)	5.9 (5.0–7.2)	<0.001
hsCRP mg/dL	0.11 (0.06–0.25)	0.11 (0.06–0.24)	0.14 (0.07–0.30)	<0.001
Height cm	165 (161–169)	165 (161–169)	164 (160–169)	0.043
Weight kg	67 (60–76)	66 (60–76)	70 (60–82)	<0.001
BMI kg/m^2^	25 (22–28)	24 (22–28)	26 (23–30)	<0.001
Obesity vs. Non-obese				<0.001
BMI < 30	83% (4003)	85% (3623)	72% (380)	
BMI ≥ 30	17% (812)	15% (661)	28% (151)	
Abdom. circumference cm	87 (79–96)	86 (79–95)	90 (80–101)	<0.001
Self-reported				
Dyslipidemia	10% (481)	9% (392)	17% (89)	<0.001
Diabetes Mellitus type 2	2% (105)	2% (87)	3% (18)	0.042
Hypertension	18% (866)	17% (729)	26% (137)	<0.001
Coronary artery disease	1% (40)	1% (32)	2% (8)	0.068
Chronic heart failure	0% (16)	0% (16)	0% (0)	0.16
Peripheral vascular disease	0% (13)	0% (12)	0% (1)	0.70
COPD	1% (67)	1% (53)	3% (14)	0.009
Metabolic syndrome ^1^	13% (602)	12% (501)	19% (101)	<0.001
SCORE2 10-yr CVD risk (%)	3 (1–5)	3 (1–5)	3 (2–5)	0.003
HbA1c in DM range				0.66
HbA1c < 6.5	99% (4526)	99% (4022)	99% (504)	
HbA1c ≥ 6.5	1% (64)	1% (58)	1% (6)	
Fasting glucose				0.71
Glucose < 126 mg/dL	98% (4703)	98% (4190)	98% (513)	
Glucose ≥ 126/dL	2% (82)	2% (72)	2% (10)	
Alcohol g/week	63 (52–76)	62 (51–75)	65 (54–78)	<0.001
Excessive alcohol intake ^2^	3% (139)	3% (120)	4% (19)	0.24
Smoking history				<0.001
Never smoker	48% (2297)	48% (2070)	43% (227)	
Previous smoking	33% (1597)	34% (1439)	30% (158)	
Current smoker	19% (925)	18% (779)	27% (146)	
Monthly household income				<0.001
≤EUR 1000	11% (509)	10% (421)	17% (88)	
EUR 1001–2000	40% (1906)	39% (1693)	40% (213)	
EUR 2001–3000	21% (995)	21% (899)	18% (96)	
EUR 3001–4000	9% (444)	9% (396)	9% (48)	
EUR 4001–5000	6% (273)	6% (264)	2% (9)	
>EUR 5000	4% (173)	4% (158)	3% (15)	
Did not disclose	11% (519)	11% (457)	12% (62)	
GISCED educational status				<0.001
Low	9% (434)	8% (356)	15% (78)	
Medium	69% (3264)	69% (2917)	66% (347)	
High	22% (1024)	22% (927)	19% (97)	

^1^ Metabolic syndrome according to International Diabetes Federation Criteria; ^2^ WHO Criteria.

**Table 3:** Logistic regression analysis: association between plaque presence and elevated BDI.

Odds Ratio (OR), adj. Relative Risk (ARR) and 95% Confidence Interval		*p*-Value	Description
Model 1	OR 1.16 (1.00–1.34)	0.043	Baseline (BDI ≥ 14)
	ARR 1.09 (1.00–1.19)	0.046	
Model 2	OR 1.43 (1.22–1.69)	<0.001	Age and sex adjusted
	ARR 1.18 (1.10–1.26)	<0.001	
Model 3	OR 1.32 (1.11–1.56)	<0.001	Age, sex, MS, and GISCED adjusted ^1^
	ARR 1.13 (1.05–1.21)	0.001	
Model 4	OR 1.21 (1.03–1.43)	0.023	Adjusted for SCORE2 components
	ARR 1.09 (1.01–1.18)	0.021	
Model 5	OR 1.25 (1.06–1.49)	0.009	Age, sex, MS, GISCED, and Med adjusted ^2^
	ARR 1.10 (1.03–1.19)	0.009	
Sensitivity Analysis 1	1.28 (1.06–1.54)	0.012	Baseline, women only
	1.25 (1.00–1.56)	0.051	Baseline, men only
Sensitivity Analysis 2	1.46 (1.17–1.81)	0.001	Baseline, age ≤ 55 years
	1.10 (0.89–1.36)	0.385	Baseline, age > 55 years

^1^ Adjusted for age, sex, metabolic syndrome (IDF criteria), and educational status (GISCED). ^2^ Model 3 with further adjustment for reported prescription of lipid-lowering medication.

**Table 4:** Logistic regression analysis: association between plaque presence per additional BDI point.

Odds Ratio (95% Confidence Interval)		*p*-Value	Description
Model 1	1.01 (1.00–1.02)	0.001	Baseline (BDI ≥ 14)
Model 2	1.02 (1.01–1.03)	<0.001	Age and sex adjusted
Model 3	1.02 (1.01–1.02)	<0.001	Age, sex, MS, and GISCED adjusted ^1^
Model 4	1.01 (1.00–1.02)	0.001	Adjusted for SCORE2 components

^1^ Adjusted for age, sex, metabolic syndrome (IDF criteria), and educational status (GISCED).

**Table 5:** Association by carotid pathology.

**Men (N = 4531)**	**Total**	**BDI ≤ 13**	**BDI ≥ 14**	***p*-Value**
Plaque (Binomial)				0.050
No Plaques	53% (2397)	53% (2239)	48% (158)	
Plaques	47% (2134)	47% (1961)	52% (173)	
Plaque diameter (cm^2^)	0.00 (0.00–18.66)	0.00 (0.00–18.57)	4.77 (0.00–20.82)	0.087
Stenosis by category				0.025
No stenosis	67% (3035)	68% (2832)	62% (203)	
ECST < 50%	32% (1448)	32% (1329)	36% (119)	
ECST 50–69%	1% (27)	1% (22)	2% (5)	
ECST 70–80%	0% (6)	0% (5)	0% (1)	
ECST > 80%	0% (4)	0% (3)	0% (1)	
**Women (N = 4819)**	**Total**	**BDI ≤ 13**	**BDI ≥ 14**	***p*-value**
Plaque (Binomial)				0.012
No Plaques	70% (3368)	70% (3022)	65% (346)	
Plaques	30% (1451)	30% (1266)	35% (185)	
Plaque diameter (cm^2^)	0.00 (0.00–5.24)	0.00 (0.00–4.98)	0.00 (0.00–6.80)	0.023
Stenosis by category				0.54
No stenosis	81% (3907)	81% (3492)	78% (415)	
ECST < 50%	19% (899)	18% (786)	21% (113)	
ECST 50–69%	0% (9)	0% (8)	0% (1)	
ECST 70–80%	0% (1)	0% (1)	0% (0)	
ECST > 80%	0% (1)	0% (1)	0% (0)	

## Data Availability

The data presented in this study are available on request from Dr. Vanessa Frey (v.frey@salk.at) pending case by case approval by the legal department of the University Hospital Salzburg to ensure that the legal requirements for data protection under the interpretation of EU and Austrian law relating to sensitive healthcare data can be met.
